# Conditioned pain modulation (CPM) paradigm type affects its sensitivity as a biomarker of fibromyalgia

**DOI:** 10.1038/s41598-024-58079-7

**Published:** 2024-04-02

**Authors:** A. Gil-Ugidos, A. Vázquez-Millán, N. Samartin-Veiga, M. T. Carrillo-de-la-Peña

**Affiliations:** Department of Clinical Psychology and Psychobiology, Brain and Pain Lab, University of Santiago de Compostela, Santiago de Compostela, Spain

**Keywords:** Conditioned pain modulation, Fibromyalgia, Chronic pain, Chronic pain, Diagnostic markers

## Abstract

Fibromyalgia (FM) is a widespread chronic pain syndrome, possibly associated with the presence of central dysfunction in descending pain inhibition pathways. Conditioned Pain Modulation (CPM) has been proposed as a biomarker of FM. Nonetheless, the wide variety of methods used to measure CPM has hampered robust conclusions being reached. To clarify the validity of CPM as a biomarker of FM, we tested two CPM paradigms (parallel and sequential) in a sample of 23 female patients and 23 healthy women by applying test (mechanical) stimuli and conditioning (pressure cuff) stimuli. We evaluated whether CPM indices could correctly classify patients and controls, and we also determined the correlations between the indices and clinical variables such as symptomatology, disease impact, depression, quality of life, pain intensity, pain interference, fatigue and numbness. In addition, we compared the clinical status of CPM responders (efficient pain inhibitory mechanism) and non-responders. We observed that only parallel CPM testing correctly classified about 70% of patients with FM. In addition, more than 80% of healthy participants were found to be responders, while the rate was about 50% in the FM patients. The sequential CPM test was not as sensitive, with a decrease of up to 40% in the response rate for both groups. On the other hand, we did not observe any correlation between CPM measures and clinical symptoms. In summary, our findings demonstrate the influence of the CPM paradigm used and confirm that CPM may be a useful marker to complement FM diagnosis. However, the findings also cast doubts on the sensitivity of CPM as a marker of pain severity in FM.

## Introduction

Fibromyalgia (FM) is defined as a widespread chronic pain syndrome associated with fatigue, sleep disturbance, cognitive impairment, affective disturbance and somatic symptoms^1^. Although the aetiology of FM remains unknown, the presence of central dysfunction in descending pain inhibition pathways is currently the best-supported hypothesis regarding the pathophysiology of the condition^2^. One of these abnormalities refers to a deficiency in conditioned pain modulation (CPM^3^). CPM occurs when the presence of a second noxious stimulus (i.e. a conditioning stimulus; CS) produces a decrease in the perceived pain evoked by a given stimulus (i.e. test stimulus; TS), applied in a contralateral area^4^. This phenomenon implies that the processing of pain signals inhibits the nociceptive input originating from a heterotopic region; deficits in the CPM mechanism could thus lead to an enhanced sensation of pain. Considered a spinal-level mediated process involving cortical regions and brainstem structures such as the anterior cingulate cortex and the periacueductal gray^5^, CPM seems to be partly mediated by diffuse noxious inhibitory controls (DNIC^6^), as demonstrated in animal studies^7,8^. Thus, descending pain-inhibitory networks play a modulatory role in CPM^9^, and disruptions in this circuitry may be associated with increased pain sensitivity^10^.

Impairments in CPM are present in several chronic pain states^11,12^, and they are particularly relevant and consistent in patients with FM^13–15^. Thus, CPM has been suggested as a potential biomarker to distinguish clinical profiles with different disease prognosis and symptom severity in chronic pain diseases^16^. For instance, Gerhardt et al.^14^ reported a significant negative correlation between CPM measures and pain intensity during the previous month in a sample of FM patients; other authors found that FM patients with defective CPM reported poorer sleep quality and showed greater impairment in sustained attention^17,18^. However, a recent review brings into question the validity of CPM as a biomarker of clinical pain^19^, given that the deficits in CPM are not always correlated with clinical manifestations of pain (pain intensity, disability due to pain, pain duration or number of painful areas). The disparity of results may be due to the heterogeneity of CPM protocols: the characteristics of the various stimuli used (e.g. CS such as cold water, tourniquets and hot water, and TS such as pressure, thermal, mechanical and electrical stimuli); the body area stimulated; the nature of stimulation (both painful and non-painful); and the mode of presentation of CS, which can be applied simultaneously or subsequently to the TS^19^. Previous research along this line has suggested that some methodological factors could influence the magnitude of the observed CPM response^20^, although the data are variable. For instance, the role of the moment of application of the CS stimulus on CPM is unclear: in some studies, the CPM response was better when the TS and CS were presented simultaneously than when they were presented sequentially^20^, while others did not find any significant differences in relation to the presentation (sequential or parallel)^21^.

Bearing this in mind, the aim of the present study was to test the validity of CPM as a diagnostic biomarker of FM, using two paradigms which differed in CS was presented either during (parallel CPM) or after the TS (sequential CPM). To this end, we compared the ability of both CPM paradigms to classify the participants as FM patients or healthy controls. As a second objective, we investigated whether CPM could be used as a biomarker of pain severity in FM, correlating CPM indices with clinical variables and comparing the clinical status of CPM responders (efficient pain inhibitory mechanism) and of non-responders.

## Methods

### Participants

The sample comprised 23 female FM patients and 23 healthy female controls. The inclusion criteria for the group of FM patients were as follows: (1) age more than 18 years; (2) diagnosis of FM by a physician or rheumatologist; and (3) fulfilment of the FM diagnosis criteria (1), i.e. a Widespread Pain Index (WPI) score of 7 or higher and a Symptom Severity Scale (SSS) score of 5 or higher or a WPI score between 3 and 6 and a SSS score of 9 or higher. Exclusion criteria were history of drug abuse and psychiatric disorders (other than depression and anxiety).

The sample size was calculated using G*Power (v.3.1.9.6.). A previous meta-analysis reported a large effect size (d = 0.78) for the difference in the efficacy of CPM between patients with chronic pain and healthy controls^12^. To be conservative, we considered a medium effect size (f = 0.25) using a repeated-measures ANOVA analysis (p < 0.05), and the sample estimation was of 44 participants (i.e., 22 participants in each group). Similar sample sizes have been reported in previous studies, from a minimum n of 10 to a maximum n of 38 in each experimental group^22^.

### CPM stimuli and procedure

*Test Stimulus (TS)*: the Pressure Pain Threshold (PPT) was obtained using an algometer (Wagner Force One, Model FDI). PPT was selected as the TS because pressure stimuli are considered more reliable than other stimuli such as heat pain thresholds^23^. Moreover, the CPM effect is best interpreted using pain thresholds and stimuli of predefined intensity as TS^24^. The area of stimulation was a 1 cm^2^ patch on the dominant forearm, over the extensor carpi radialis longus, and the pressure velocity (rate) was 35 kPa/s. Given the precision required, the test was carried out by a trained investigator capable of keeping the rate within the required rate. The stimulation was delivered 3 times, separated by 20 s, and the mean value was considered the PPT.

*Conditioning stimulus (CS)* was delivered by a pressure cuff on the opposite arm, with a constant pressure of 240 mm hg (30 kPa), applied for 120 s. Previous data supports that using the contralateral dermatome provides the most reliable results^25^.

**Figure 1:**
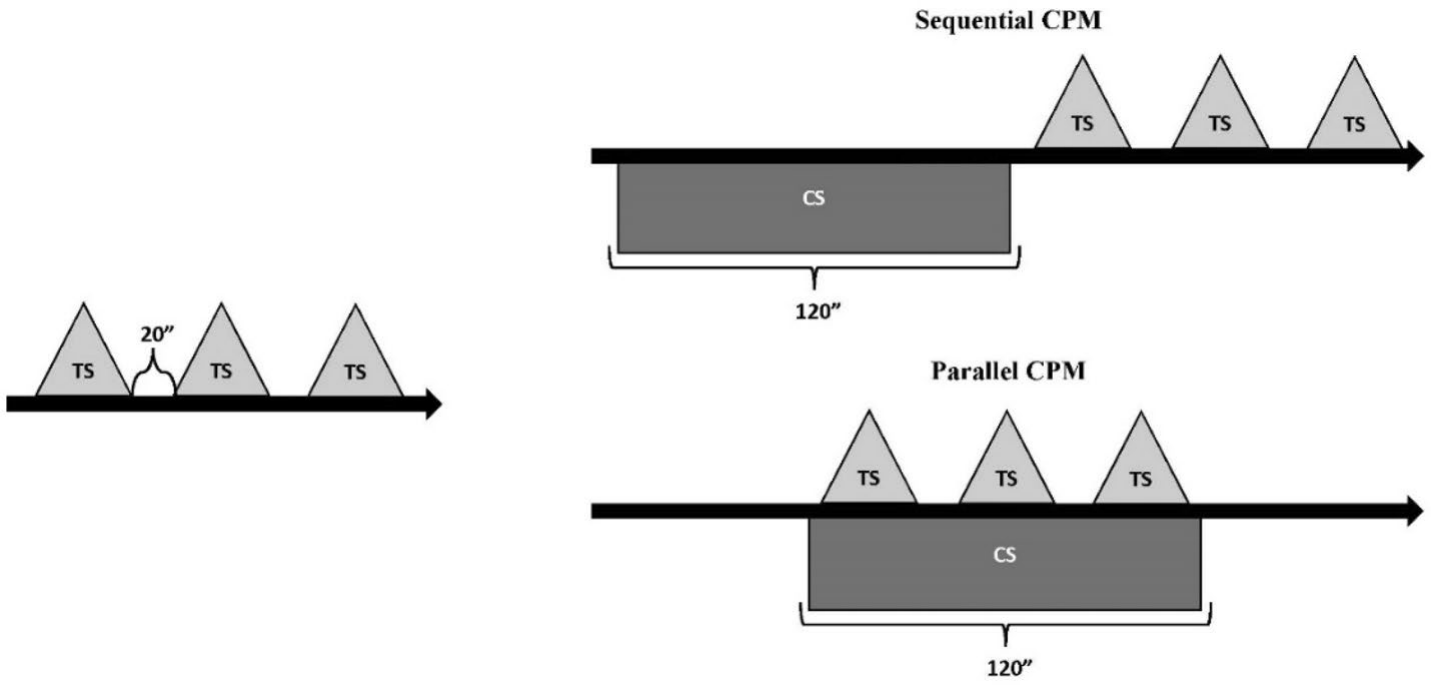
CPM procedures used in the study. All the participants underwent both paradigms in counterbalanced order.

Two paradigms were delivered for all the participants, in a counterbalanced order with an inter-protocol interval of 10 min (see Fig. 1):

*Sequential paradigm*: The TS (pressure algometer) was delivered before the CS (pressure cuff) and after the CS (mean of 3 PPT measurements separated by 20 s).

*Parallel paradigm*: The TS was assessed before and during the application of the CS (pressure cuff).

*Calculation of the CPM effect*: We calculated the CPM effect for each experimental paradigm by calculating the mean PPT for TS after/during presentation of the CS (either sequential or parallel) minus the mean PPT for TS before the CS. Positive values (CPM > 0) indicated an elevation of the threshold, i.e. an inhibitory response due to application of the CS or more efficient CPM.

### Questionaire measurement

Both FM patients and healthy participants completed different validated Spanish versions of tests and questionnaires of interest to the study:

The Fibromyalgia Survey Questionnaire (FSQ^26^) was used to assess FM symptoms, to check the inclusion criteria for the patients and characterize the groups. The FSQ is based on the diagnostic criteria proposed by Wolfe et al.^1^, and includes the Symptom Severity Scale (SSS) and the Widespread Pain Index (WPI). The SSS considers three key symptoms (fatigue; cognitive problems in attention, concentration or memory; and non-restorative sleep), assessed on a scale of 0–3 (0 = not present to 3 = extreme). In addition, the SSS assesses abdominal pain, depression and headache, determined as present (1) or not present (0). The SSS score ranges from 0 to 12. The WPI score indicates the number of body areas with pain reported by the patient (from 0 to 19).

The Beck Depression Inventory (BDI-1A^27^) was used to evaluate the severity of depressive symptoms. The BDI-1A is composed by 21 items representative of symptoms such as sadness, feelings of failure, pessimism, suicidal desire, etc. Each item is answered by the participants on a 4-point scale, ranging from 0 to 3. The total score (range from 0 to 63) was recorded. Higher scores are associated with greater severity of depressive symptoms, and patients can be classified as having no depression (0–13), mild depression (14–19), moderate depression (20–28) or severe depression (29–63).

The Revised Fibromyalgia Impact Questionnaire (FIQ-R^28^) was administered to measure the functional disability and health status of patients with FM. The FIQ-R is a self-reporting questionnaire including 21 items scored on an 11-point numerical rating scale of 0–10, with 10 being “worst.” The scores are calculated for three domains: function (from 0 to 30), general impact (from 0 to 20) and symptoms (from 0 to 50). A total score (range from 0 to 100) was also considered. Higher scores are associated with greater disease severity and functional impact, and patients are classified as having mild (0–38), moderate (39–58) or severe (59–100) symptoms.

The 36-item Short Form Health Survey (SF-36^29^) assesses Quality of Life (QoL) and provides a profile of health status and function. It is composed of 36 items distributed across eight scales: physical function, physical role, body pain, general health, vitality, social function, emotional role and mental health (i.e. the most relevant health concepts included in the Medical Outcomes Study (MOS)). The scores on each subscale range from 0 to 100 (0 represents the worst possible health level and 100, the best). In this study, we calculated the score for the eight subscales and a mean score for the SF-36.

In addition, participants completed different ad hoc Visual Analogue Scales (VAS) related to their status in the last week (pain intensity, numbness, fatigue), in the last month (pain intensity, interference due to pain, depressive state) and the level of deterioration in their health.

### Data analysis

The pretest clinical status of the clinical and control groups was first compared. As none of the variables studied (except age) met the criteria for normality in both groups (based on Kolmogorov–Smirnov and Shapiro–Wilk test), non-parametric tests were used to calculate differences in the mean values (Man Whitney U test).

Repeated-measures ANOVA was then used to compare the CPM results obtained by the groups. Paradigm (levels: sequential and parallel) was used as an intra-subject factor and Group (levels: FM patients and healthy controls) as an inter-subject factor.

The patients were then classified as responders or non-responders by both paradigms. For a patient to be considered a CPM responder, the difference between the PPT to the TS before and after the CS had to be greater than the standard deviation for the mean of the group (CPM Effect > PPT SD). Algometry has an intrinsic standard error of measurement associated with the assessment procedure itself, just as all psychophysical methods^30,31^. Thus, the rationale behind our classification procedure was to ensure that the differences found in pain thresholds before and after the CS were not due to the standard error, but to the application of the second noxious stimuli. After being classified, the number of responders for each group in each of the paradigms was compared using chi-square tests.

Binary logistic regression analysis was used to assess the sensitivity and specificity of the parallel CPM and sequential CPM paradigms to correctly classify the participants as FM patients or healthy controls.

Finally, considering all of the clinical variables, we performed non-parametric tests (Man Whitney U) to check possible differences in the clinical profile of FM patients who were CPM responders and those who were CPM non-responders. The Bonferroni-Holm correction for multiple comparisons was performed. Also, we calculated Pearsons’ r coefficients to explore possible correlation between CPM magnitude and clinical variables.

Data were analyzed using the SPSS statistical package (v.24.0; IBM Corporation, Armonk, NY, United States) and JAPS (v.0.18; The JASP Team).

### Ethics statement

The study involves human participants, followed the Declaration of Helsinki and was reviewed and approved by the Research Ethics Committee of Galicia (CEIC-SERGAS; code: 2021/021). The participants provided their written informed consent to participate in this study.

## Results

### Sample characteristics and baseline scores

Participants in both groups were matched by age (patients: mean 49.78 years (± 8.67); healthy controls: mean 50.22 years (± 12.57); *t* = 0.137; *p* = 0.892) and menstrual phase (*X*^*2*^ = 1.575; *p* = 0.665).

The *Man Whitney* U test results showed that patients with FM obtained lower scores than the healthy controls for all the clinical variables. Thus, patients with FM had a higher rate of depressive symptoms, poorer quality of life, fatigue state and numbness, greater interference in their daily life due to pain and greater pain intensity, both in the previous week and in the previous month (see Table 1). The PPTs were similar in both groups.

**Table 1:** Comparison between groups in the variables assessed.

	FM patients mean (SD)	Healthy controls mean (SD)	Man-ehitney-U Z score	Sig
BDI	15.52 (± 6.74)	4.22 (± 6.05)	− 4.889	*p* < 0.001
FIQ-R	47.71 (± 24.14)	5.95 (± 5.17)	− 5.636	*p* < 0.001
SSS	7.57 (± 1.97)	2.87 (± 1.46)	− 5.621	*p* < 0.001
WPI	9.26 (4.63)	2.35 (± 1.64)	− 4.816	*p* < 0.001
SF-36	48.07 (± 15.37)	75.73 (± 9.06)	− 5.042	*p* < 0.001
Intensity of fatigue -previous week (VAS)	6.7 (± 1.94)	2.26 (± 1.42)	− 5.310	*p* < 0.001
Intensity of numbness-previous week (VAS)	6.22 (± 3.72)	0.35 (± 0.57)	− 5.150	*p* < 0.001
Intensity of pain-previous week (VAS)	5.43 (± 2.84)	1.87 (± 1.01)	− 4.344	*p* < 0.001
Intensity of pain-previous month (VAS)	5.65 (± 2.69)	2.22 (± 1.09)	− 4.413	*p* < 0.001
PPT (Kgf/cm^2^)	1.27 (± 0.68)	1.27 (± 0.26)	− 0.616	*p* = 0.538
Sequential CPM (Kgf/cm^2^)	− 0.004 (± 0.41)	0.039 (± 0.31)	− 0.155	p = 0.877
Parallel CPM (Kgf/cm^2^)	0.3 (± 0.56)	0.87 (± 0.63)	− 2.874	p = 0.004

*BDI* Beck depression inventory, *FIQ-R* Fibromyalgia impact questionnaire -revised; *SSS* Symptom severity scale, *WPI* Widespread pain index, SF36: Short form health survey, *VAS* Visual analogue scale, *PPT* Pressure pain threshold, measured in kgf/cm2.

*CPM* Conditioned Pain Modulation (subtraction of mean PPT to TS after(sequential)/during (parallel) the presentation of the CS minus the mean PPT to TS before CS. Smaller CPM indices are indicative of less efficient pain modulation mechanisms).

### CPM effect: modulation by paradigm

**Figure 2:**
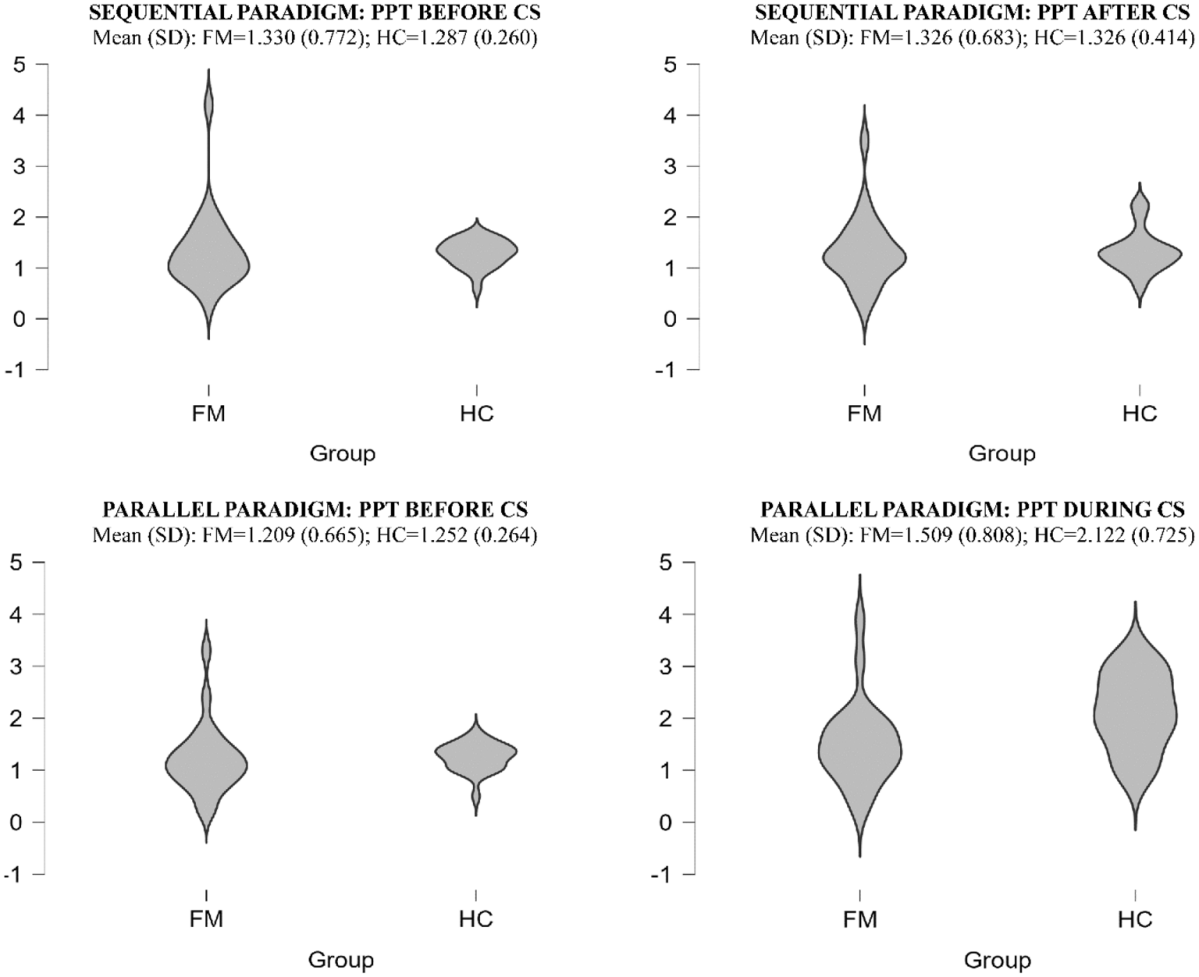
Violin plots for the PPT to TS measurements taken before and after/during the CS in each of the CPM paradigms. (*CS* Conditioning stimulus, *PPT* Pressure Pain Threshold, *FM* Patients with Fibromyalgia, *HC* Healthy controls). As may be seen, the HCs showed a clear CPM effect in the parallel paradigm.

The repeated measures ANOVA showed a significant main effect of Paradigm (F (1,44)  = 29.513; p < 0.001), as well as a Paradigm x Group interaction (F (1,44)  = 6.343; p = 0.015). Post-hoc tests (with Bonferroni correction) showed that the parallel CPM produced more effective inhibitory responses (i.e. higher thresholds to the second application of the TS; Parallel CPM =  0.59; Sequential CPM =  0.02; p < 0.001), while the differences between the FM patients and healthy controls were only significant for the parallel CPM (FM patients =  0.30; Healthy controls: 0.87; p = 0.004). The differences in the inhibitory effect achieved in both paradigms are clearly shown in Fig. 2.

### Distribution of CPM responders and non-responders in each group

The participants were classified as CPM responders and CPM non-responders (see Table 2). Application of the sequential paradigm yielded a similar number of responders in the patients and control group (according to the chi-square test results). However, application of the parallel paradigm yielded a significantly greater number of healthy controls with an adequate CPM response than of FM patients (X^2^ = 3.860; p = 0.049).

**Table 2:** Distribution of responders and non-responders in the different paradigms and groups.

	Sequential CPM		Parallel CPM	
	CPM Responders	CPM non-responders	CPM Responders	CPM Non-responders
FM patients (N = 23)	7 (30.43%)	16 (69.67%)	13 (56.52%)	10 (43.48%)
Healthy controls (N = 23)	9 (39.13%)	14 (61.87%)	20 (86.96%)	3 (13.04%)
Total	16	30	33	13
	*X*^*2*^ = 0.096 (*p* = 0.757)		**X**^**2**^** = 3.860 (*p* = 0.049)**	

Participants were classified as responders if the magnitude of the CPM (difference of PPT before vs. after the CS) was greater than the standard deviation for the mean of the group (CPM > SD).

*CPM* Conditioned Pain Modulation.

Significant values are in bold.

### Accuracy of CPM for classifiying healthy controls and FM patients

In the binary logistic regression model including the effect of the parallel CPM paradigm as the predictor variable, the Omnibus test yielded *X*^*2*^ = 9.569 (*p* = 0.002) so the inclusion of this variable contributes to explaining the group to which each participant belongs.

Given this effect, the model correctly classified 69.6% of the participants as patients or controls (see Table 3). The Nagelkerke R^2^ index suggests a moderate relationship between the predictor and the outcome.

**Table 3:** Binary logistic regression model results using the parallel CPM paradigm for predicting and classifying participants.

		Predicted		
		FM patients	Healthy controls	Correctly classified
Observed	FM patients	16	7	69.6%
	Healthy controls	7	16	69.6%

β	Odds ratio	Sig	Cox and Snell R^2^	Nagelkerke R^2^
− 1.593	0.203	*p* = 0.006**	0.188	0.250

**Figure 3:**
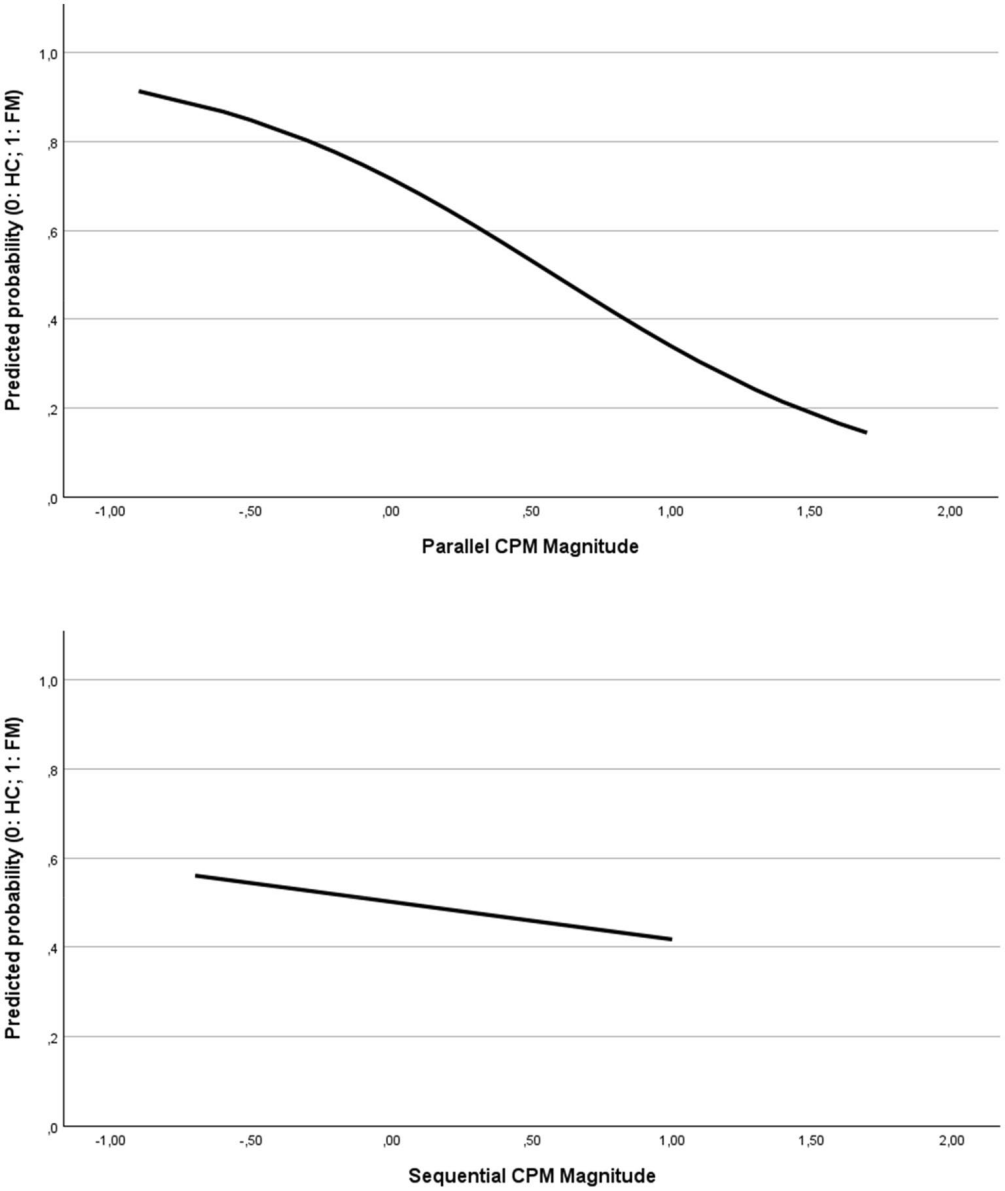
Results of the binary logistic regression model for the parallel CPM (upper area) and sequential CPM (lower area). The curve represents the probability of being classified as a patient according to the magnitude of CPM. For the parallel CPM, the smaller the magnitude of CPM, the higher the probability of belonging to the FM group. Sequential CPM did not significantly classify HC vs. FM.

On the contrary, the result for the sequential CPM was nonsignificant (p = 0.707) (see Fig. 3).

### Differences in the clinical profile of CPM responder and non-responder patients

For the sample of patients, non-parametric tests revealed significant differences between CPM responder and CPM non-responder patients in indices of disease severity, assessed by the FIQ-R: patients who exhibited an efficient CPM response had less severe symptoms (only using data from the parallel paradigm). A significant difference in the WPI index (body extent of pain) was also found, although in the opposite direction: CPM responder patients showed more generalized pain throughout the body than their non-responsive counterparts (see Table 4). However, after applying the Bonferroni-Holm correction for multiple comparisons, none of the clinical variables maintained their significance level (except for the CPM effect itself).

**Table 4:** Clinical state differences between parallel-CPM responders and non-responders (only data for the parallel paradigm are shown).

	CPM responder (N = 13)	CPM Non responder (N = 10)	*Mann–whitney-U Z score*	Sig
BDI	13.85 (± 6.19)	17.70 (± 7.12)	− 1.123	0.261
FIQ-R	38.12 (± 18.03)	60.18 (± 26.18)	− 1.985	0.047*
SSS	6.85 (± 1.41)	8.50 (± 2.32)	− 1.766	0.077
WPI	11.08 (± 4.15)	6.90 (± 4.31)	− 2.030	0.042*
SF-36	52.26 (± 13.21)	42.64 (± 16.93)	− 1.488	0.137
Intensity of fatigue-previous week (VAS)	6.23 (± 1.69)	7.30 (± 2.16)	− 1.923	0.055
Intensity of numbness—previous week (VAS)	6.08 (± 3.57)	6.40 (± 4.09)	− 0.317	0.751
Intensity of pain- previous week (VAS)	4.77 (± 2.49)	6.30 (± 3.16)	− 1.467	0.142
Intensity of pain- previous month (VAS)	5.15 (± 1.19)	6.30 (± 3.23)	− 1.407	0.159
PPT (Kgf/cm2)	1.22 (± 0.86)	1.33 (± 0.38)	− 1.430	0.153
CPM (Kgf/cm2)	0.64 (± 0.42)	-0.14 (± 0.41)	− 3.522	** < 0.001**

*BDI *Beck depression inventory*, FIQ-R *Fibromyalgia impact questionnaire –revised, *SSS *Symptom severity* Scale*, *WPI: Widespread Pain Index; SF36 *Short form health survey, *VAS* Visual analogue scale, *PPT *Pressure pain threshold*, measured in kgf/cm2.*

*The significance level was not maintained after Bonferroni-Holm correction. Significant values are in bold.

### Correlation between CPM effect and clinical variables

Analysis of the correlation between the clinical variables evaluated and the sequential or parallel CPM indices did not indicate the presence of any significant correlation (see Table 5).

**Table 5:** Correlation between clinical variables and CPM results in the clinical group (n = 23).

	Sequential CPM		Parallel CPM	
	Pearson r coefficient	Sig	Pearson r coefficient	Sig
BDI	− 0.294	0.173	0.176	0.423
FIQ-R	0.076	0.730	0.217	0.320
SSS	− 0.135	0.539	0.161	0.462
WPI	0.137	0.534	-0.290	0.179
SF36	− 0.102	0.644	0.069	0.753
Intensity of fatigue-previous week (VAS)	− 0.360	0.091	0.336	0.117
Intensity of numbness-previous week (VAS)	− 0.234	0.282	0.007	0.977
Intensity of pain-previous week (VAS)	− 0.152	0.488	0.250	0.251
Intensity of pain-previous month (VAS)	− 0.227	0.297	0.114	0.605
Pain pressure threshold (PPT)	0.249	0.252	0.092	0.677

*BDI *Beck depression inventory, *FIQ-R* Fibromyalgia impact questionnaire-revised*, SSS* Symptom severity scale, *WPI* Widespread pain index,* SF36 *Short form health survey,* VAS* Visual analogue scale*, PPT* Pressure pain threshold, measured in kgf/cm^2^.

## Discussion

Application of a heterotopic noxious stimulus (Conditioning Stimuli-CS) provokes a reduction in pain sensitivity (widely referred as the CPM effect) and this mechanism of pain modulation is known to be impaired in chronic pain populations^32^. Thus, the use of a CPM index^3^ has been suggested as a potential biomarker to distinguish clinical profiles in different chronic pain syndromes, such as Fibromyalgia (FM) (16; 15).

Although conceptually clear, the wide variety of the procedures used in CPM testing makes it difficult to compare results across studies and to obtain solid conclusions about the utility of CPM as a biomarker of chronic pain^19^. To date, the stimuli most frequently used as Test Stimuli (TS) have been mechanical stimuli such as pressure pain produced by an algometer^33^ or tourniquet^34^, electrical stimuli^35^ or thermal stimuli delivered with thermodes^36^. Similar variability can be found for the CS, with immersion in cold water^37^ and tourniquet pressure^38^ being the most common stimuli. Previous CPM studies have used a combination of stimuli, with different duration, location, temporality and intensity, and with the experimental design being adapted to their interests. Consequently, there is a lack of a standardized method for CPM testing^25^.

In this study, we assessed the validity of two CPM paradigms (parallel and sequential) as diagnostic biomarkers of FM, by comparing patients and healthy controls. Considering that the reliability of the CPM paradigm is highly dependent on the chosen stimulus combination and characteristics^39^, we used mechanical stimuli, delivered by an algometer (TS) and a pressure cuff (CS), as these produced good results in a pilot study. Participants in both groups were classified as CPM responders and non-responders.

When the parallel paradigm was used, more than 80% of healthy participants were classified as responders, while in the FM patients the rate was about 50%. This difference seems to reinforce the widely reported presence of a CPM impairment in patients with FM and other chronic pain conditions^12–15^, which may be due to impaired descending inhibitory pain modulation^40^. We also found that parallel CPM testing correctly classified almost 70% of patients with FM. On the other hand, when the sequential paradigm was used, the rate of responders in both patients and healthy participants was less than 40%, with no difference in the between-group analysis. Thus, the parallel protocol appears to generate a more consistent CPM effect. Likewise, different studies have found that the sequential paradigm was less efficient in different pain conditions like migraine^41^. Thus, our findings confirm the utility of CPM for classifying FM patients and healthy controls, although only the parallel CPM paradigm proved useful for classification. This demonstrates that some methodological factors can influence the magnitude of the CPM effect and its clinical application^20,39,42^, and it reinforces the need for a standardized protocol.

Our results are consistent with those of previous studies conducted in patients with FM, highlighting the existing dysfunction in CPM in these patients. However, most previous FM studies have obtained similar results with sequential paradigms. For example, in a recent study, Knezevic and cols^23^ reported lower pain thresholds and clear impairment of CPM in FM patients. Previous studies also observed lower CPM inhibitory efficacy in FM, although it is not clear whether the impaired endogenous pain inhibition is a cause or consequence of prolonged pain^43^. Given the effectiveness of the sequential paradigm in these studies and as the differences between the sequential and parallel protocols may not be significant^21^, the fact that we observed that the parallel paradigm was much superior is of interest. Considering that different chronic pain pathologies like knee osteoarthritis^44,45^, migraine^20^ and lumbosacral radiculopathy^46^ cause alterations in the CPM effect, it would be interesting to further investigate several pain pathologies using different protocols and combinations of stimuli to identify the typical patterns or profiles for each pathology. The final goal is to develop a standardized CPM protocol, which could be adapted to each of the most prevalent chronic pain pathologies.

There is a consistent body of literature supporting the utility of CPM for classifying patients with chronic pain problems and as a marker for diagnosis, prognosis and treatment of different diseases. CPM tests can improve the determination of different pain modulation profiles^47^. Furthermore, considering whether a given person has a pronociceptive or antinociceptive profile makes it easier to predict the future occurrence of pain and to make decisions about treatment^48^. In this vein, there is evidence for the utility of CPM to predict surgery outcomes: patients who exhibited a dysfunctional endogenous pain modulatory system prior to surgery were found to be at greater risk of developing chronic pain postoperatively^37^. This study shows that the parallel CMP can correctly classify 70% of the patients, and thus supports its use as a complementary method for diagnosing FM.

Diagnosis, understanding of pathophysiological pathways and treatment of FM have been hampered by the lack of blood biomarkers of the disease and the subjective nature of the pain experience. The incorporation of potential biomarkers based on the objective measurement of pain thresholds and endogenous central mechanisms of analgesia is crucial to advance the understanding of FM^49,50^. They allow for the generation or validation of classification criteria, and even help to improve the design of protocols to develop other treatment modes. As an example, pharmacological trials to test new drugs for pain treatment can be improved if CPM is used for enriched enrolment. CPM identifies individuals with faulty pain modulation mechanisms, and if enrolled selectively, can increase the chances of better results, and optimise the clinical trial.

Nevertheless, some inconsistent results were obtained regarding the clinical relevance of CPM and its usefulness as a marker of disease severity. We did not find significant differences between patients with (non-responders) and without (responders) impaired CPM as regards the severity of FM symptoms, while the CPM magnitude and scores in clinical symptoms were uncorrelated. Similarly, previous studies yielded mixed results regarding the relationship between CPM responses and the clinical characteristics of FM patients. For example, some authors found that patients with FM and defective endogenous pain modulation had poorer sleep quality and greater impairment in cognitive functioning (i.e. sustained attention) (17; 18), while others reported that the spatial extent of pain was not associated with CPM in FM patients^14^. A recent systematic review also showed no significant correlations between clinical pain (intensity, interference due to pain) and CPM^19^. In summary, the above results cast doubts about the sensitivity of CPM as a marker of disease severity, at least for now.

Several factors may have affected our results. First, it has been shown that the CPM effect is induced by the use of different combinations and types of stimuli (pressure stimuli, calorific stimuli, cold stimuli, among others). In this study, we used a combination of two pressure stimuli, but testing other combinations of stimuli and including quantitative sensory testing (QST) measures complementary to CPM, such as temporal summation (TS) or exercise-induced analgesia, could be used to better characterize both pronociceptive and antinociceptive mechanisms in patients with FM.

On the other hand, we did not control the effects of inter- and intra-individual variability on CPM. Previous studies have reported that individual characteristics such as age^51^, sex^51^, alcoholism^52^, sleep^52^, socioeconomic status^51^, exercise^51^, ovulatory phase ^53^, education level^54^, psychological factors^21^ and chronic stress^55^, among other, can affect the CPM response. Situational factors such as attentional focus on the CS^55^ or the patients’ expectations^21^ can also influence CPM. We used a balanced sample in terms of age, but composed exclusively of women (menstrual phase controlled), to better control gender differences. Although FM is generally considered much more prevalent in women than in men^56^, this assumption is now under debate^57^. In addition, there was no control over the medication taken by the participants, which could influence the variables evaluated. Given the complexity of the FM syndrome, evaluation of larger samples and subgroup analysis are desirable. For all these reasons, the findings must be extrapolated with caution and assumptions considered carefully.

## Conclusions

FM is a widespread chronic pain syndrome of unknown etiology but often related to a central dysfunction in descending inhibitory pathways. In this study, we investigated whether CPM could be used as a sensitive biomarker of FM diagnosis and pain severity. We found that the CPM paradigm used (parallel or sequential) strongly affected the results obtained. The parallel CPM showed 70% accuracy in classifying participants as patients or controls, and it produced a higher proportion of impaired pain modulation patterns in the FM group. This result reinforces the widely reported presence of CPM impairment in FM patients but suggests the need to use a standardized method of assessment.

Concerning the sensitivity of CPM as a biomarker of pain severity in FM, we did not find any significant correlation between CPM and clinical symptoms.

Overall, our findings confirm the influence of some methodological factors on the magnitude of the CPM effect and suggest the usefulness of CPM (parallel paradigm) as a diagnostic tool for FM; however, they cast doubts on the sensitivity of CPM as a biomarker of disease severity.

## Data Availability

The raw data supporting the conclusions of this article will be made available by the authors, without undue reservation.
